# Supplementary material for: Studying and Modeling of the Extraction Properties of the Natural Deep Eutectic Solvent and Sorbitol-Based Solvents in Regard to Biologically Active Substances from Glycyrrhizae Roots
Source: Molecules. 2020 Mar 25;25(7):1482. doi: 10.3390/molecules25071482 (PMC7180746; doi:10.3390/molecules25071482)
Supplement: Supplementary file 1 [file molecules-25-01482-s001.pdf]

SUPPLEMENTARY MATERIALS

Studying and Modeling of the Extraction Properties of the Natural Deep Eutectic Solvent and Sorbitol-Based Solvents in Regard to Biologically Active Substances from *Glycyrrhizae* Roots

Nikolay Boyko [1,*], Elena Zhilyakova [1], Anastasiya Malyutina [1], Oleg Novikov [2], Dmitriy Pisarev [2], Rimma Abramovich [2], Olga Potanina [2], Simon Lazar [2], Praskovia Mizina [3] and Rita Sahaidak-Nikitiuk [4]

[1] Belgorod National Research University, BelSU, 308015 Belgorod, Russia; boykoniknik@gmail.com (N.B.), ezhilyakova@bsu.edu.ru (E.Z.), malyutina_a@bsu.edu.ru (A.M.)
[2] Peoples' Friendship University of Russia, RUDN University, 117198 Moscow, Russia; ole9222@yandex.ru (O.N.), juniper05@mail.ru (D.P.), abramovich-ra@rudn.ru (R.A.), potanina-og@rudn.ru (O.P.), sjl512am@yandex.ru (S.L.)
[3] All-Russian Scientific Research Institute of Medicinal and Aromatic Plants, ARSRIMAP, 117216 Moscow, Russia,); mizina-pg@yandex.ru
[4] National University of Pharmacy, NUPh, Kharkiv 61002, Ukraine; sagaidak_rita@ukr.net
* Correspondence: boykoniknik@gmail.com; Tel.: +7-980-326-8487

Appendix S1: The algorithm and intermediate computations of the final form of the mathematical model.

The law of statistical physic is Boltzmann law with discrete energy levels of molecules (or quantum Fermi-Dirac statistics):

$$\frac{n}{n_0}=\frac{C\cdot V}{m_0}=\frac{1}{1+\exp\left[\frac{\Delta\mu}{RT}+a\right]} \tag{1}$$

The law of thermodynamics is the change of BAS molecules' chemical potential at transition from plant raw material (phase 2) to the solvent (phase 1):

$$\Delta\mu = dG1 - dG2 + dG_{unpred} \tag{1.1}$$

where Δμ is the change of BAS molecules' chemical potential at transition from plant raw material to the solvent, J/mole; *dG*1 is Gibbs energy represented by the interaction energy between molecules of the BAS and the solvent, J/mole; *dG*2 Gibbs energy represented by the is interaction energy between molecules of the BAS molecules and the plant raw material matrix after its impregnation with the solvent, J/mole; ΔG_{unpred} are unpredicted energetically processes, J/mole.

The laws of thermodynamics and physical chemistry are Gibbs energy of BAS molecules in solvent *dG*1 that is connected with intermolecular energy (Van-der-Waals forces):

$$dG1=\frac{(e_1\cdot z_1)\cdot\mu_2}{(4\cdot\pi\cdot\varepsilon_0\cdot\varepsilon)\cdot r^2}+\frac{2\cdot{\mu_1}^2\cdot{\mu_2}^2}{3\cdot(4\cdot\pi\cdot\varepsilon_0\cdot\varepsilon)^2\cdot k\cdot T\cdot r^6}+\frac{3\cdot\alpha_1\cdot\alpha_2\cdot(I_1\cdot I_2)}{2\cdot(4\cdot\pi\cdot\varepsilon_0\cdot\varepsilon)^2\cdot(I_1+I_2)\cdot r^6} \tag{1.2}$$

And the Gibbs energy of BAS molecules in plant raw material *dG*2:

$$dG2=\frac{(e_1\cdot z_1)\cdot\mu_3}{(4\cdot\pi\cdot\varepsilon_0\cdot\varepsilon_y)\cdot r^2}+\frac{\mu_1\cdot\mu_3}{(4\cdot\pi\cdot\varepsilon_0\cdot\varepsilon_y)\cdot r^3}+\frac{3\cdot\pi\cdot n\cdot\alpha_1\cdot\alpha_3\cdot(I_1\cdot I_3)}{2\cdot 6\cdot(4\cdot\pi\cdot\varepsilon_0\cdot\varepsilon_y)^2\cdot(I_1+I_3)\cdot r^3} \tag{1.3}$$

Intermolecular energy (Ion-Dipole, Dipole-Dipole (Keesom), Dispersion (London), etc.) between the BAS (index 1) molecules and solvent molecules (index 2) in the liquid:

$$dG_{i-d}=\frac{(e_1\cdot z_1)\cdot\mu_2}{(4\cdot\pi\cdot\varepsilon_0\cdot\varepsilon)\cdot r^2} \tag{1.4}$$

$$dG_{d-d}=\frac{2\cdot{\mu_1}^2\cdot{\mu_2}^2}{3\cdot(4\cdot\pi\cdot\varepsilon_0\cdot\varepsilon)^2\cdot k\cdot T\cdot r^6} \quad \text{(Keesom in liquid)} \tag{1.5}$$

$$dG_{disp} = \frac{3 \cdot \alpha_1 \cdot \alpha_2 \cdot (I_1 \cdot I_2)}{2 \cdot (4 \cdot \pi \cdot \varepsilon_0 \cdot \varepsilon)^2 \cdot (I_1 + I_2) \cdot r^6} \quad \text{(London in liquid)} \qquad (1.6)$$

Intermolecular energy between the BAS (index 1) molecules and cellulose / lignin molecules (index 3) in plant raw material after its impregnation with the solvent:

$$dG_{i-d} = \frac{(e_1 \cdot z_1) \cdot \mu_3}{(4 \cdot \pi \cdot \varepsilon_0 \cdot \varepsilon_y) \cdot r^2} \qquad (1.7)$$

$$dG_{d-d} = \frac{\mu_1 \cdot \mu_3}{(4 \cdot \pi \cdot \varepsilon_0 \cdot \varepsilon_y) \cdot r^3} \quad \text{(Keesom in solid)} \qquad (1.8)$$

$$dG_{disp} = \frac{3 \cdot \pi \cdot n \cdot \alpha_1 \cdot \alpha_3 \cdot (I_1 \cdot I_3)}{2 \cdot 6 \cdot (4 \cdot \pi \cdot \varepsilon_0 \cdot \varepsilon_y)^2 \cdot (I_1 + I_3) \cdot r^3} \quad \text{(London in solid)} \qquad (1.9)$$

$$\varepsilon_y = \varphi \cdot \varepsilon + (1 - \varphi) \cdot \varepsilon_3 \qquad (1.10)$$

π is a mathematical constant, 3.14; ε_0 is an electrical constant, $8.85 \cdot 10^{-12}$ F/m; ε, ε_y, ε_3 are dielectric constants of the solvent; the plant raw material matrix after its impregnation with the solvent and the cellulose, respectively; μ_1, μ_2, μ_3 are molecules' dipole moments for the BAS, the solvent, and the plant raw material matrix, respectively, C·m; *n* is number of plant raw material matrix atoms per volume, units/m^3; α_1, α_2, α_3 are molecules' polarizability for the BAS, the solvent, and the plant raw material matrix, respectively, m^3; I_1, I_2, I_3 are molecules' ionization energy for the BAS, the solvent, and the plant raw material matrix, respectively, J; *r* is the distance between the molecules, m; φ is the mole fraction of the plant raw material matrix.

Due to the fact that both, Coulomb and Van-der-Waals forces (Keesom, London, etc.) are of electromagnetic nature, the authors used an assumption that the intermolecular energy equations should include the dielectric constant of the medium (solvent), in which the BAS molecules dissolve we have:

$$\Delta\mu = dG1 - dG2 \pm dG_{unpred} = \frac{(e_1 \cdot z_1) \cdot \mu_2}{(4 \cdot \pi \cdot \varepsilon_0 \cdot \varepsilon) \cdot r^2} + \frac{2 \cdot \mu_1^2 \cdot \mu_2^2}{3 \cdot (4 \cdot \pi \cdot \varepsilon_0 \cdot \varepsilon)^2 \cdot k \cdot T \cdot r^6} + \frac{3 \cdot \alpha_1 \cdot \alpha_2 \cdot (I_1 \cdot I_2)}{2 \cdot (4 \cdot \pi \cdot \varepsilon_0 \cdot \varepsilon)^2 \cdot (I_1 + I_2) \cdot r^6} - \\ - \frac{(e_1 \cdot z_1) \cdot \mu_3}{(4 \cdot \pi \cdot \varepsilon_0 \cdot \varepsilon_y) \cdot r^2} - \frac{\mu_1 \cdot \mu_3}{(4 \cdot \pi \cdot \varepsilon_0 \cdot \varepsilon_y) \cdot r^3} - \frac{3 \cdot \pi \cdot n \cdot \alpha_1 \cdot \alpha_3 \cdot (I_1 \cdot I_3)}{2 \cdot 6 \cdot (4 \cdot \pi \cdot \varepsilon_0 \cdot \varepsilon_y)^2 \cdot (I_1 + I_3) \cdot r^3} \pm dG_{unpred} \qquad (1.11)$$

$$\Delta\mu = \left(\frac{(e_1 \cdot z_1) \cdot \mu_2}{(4 \cdot \pi \cdot \varepsilon_0 \cdot \varepsilon) \cdot r^2} - \frac{(e_1 \cdot z_1) \cdot \mu_3}{(4 \cdot \pi \cdot \varepsilon_0 \cdot \varepsilon_y) \cdot r^2} - \frac{\mu_1 \cdot \mu_3}{(4 \cdot \pi \cdot \varepsilon_0 \cdot \varepsilon_y) \cdot r^3} \right) + \\ + \left(\begin{matrix} \frac{2 \cdot \mu_1^2 \cdot \mu_2^2}{3 \cdot (4 \cdot \pi \cdot \varepsilon_0 \cdot \varepsilon)^2 \cdot k \cdot T \cdot r^6} + \frac{3 \cdot \alpha_1 \cdot \alpha_2 \cdot (I_1 \cdot I_2)}{2 \cdot (4 \cdot \pi \cdot \varepsilon_0 \cdot \varepsilon)^2 \cdot (I_1 + I_2) \cdot r^6} - \\ - \frac{3 \cdot \pi \cdot n \cdot \alpha_1 \cdot \alpha_3 \cdot (I_1 \cdot I_3)}{2 \cdot 6 \cdot (4 \cdot \pi \cdot \varepsilon_0 \cdot \varepsilon_y)^2 \cdot (I_1 + I_3) \cdot r^3} \end{matrix} \right) \pm dG_{unpred} \qquad (1.12)$$

$$\Delta\mu = \left(\frac{A}{\varepsilon} - \frac{B}{\varepsilon_y} \right) + \left(\frac{C}{\varepsilon^2} - \frac{D}{\varepsilon_y^2} \right) \pm dG_{unpred} = \frac{1}{\varepsilon^2} \cdot \left(C - \frac{\varepsilon^2}{\varepsilon_y^2} \cdot D \right) + \frac{1}{\varepsilon} \left(A - \frac{\varepsilon}{\varepsilon_y} \cdot B \right) \pm dG_{unpred} \qquad (1.13)$$

where $A = \frac{(e_1 \cdot z_1) \cdot \mu_2}{(4 \cdot \pi \cdot \varepsilon_0) \cdot r^2}$; $B = \left(\frac{(e_1 \cdot z_1) \cdot \mu_3}{(4 \cdot \pi \cdot \varepsilon_0) \cdot r^2} + \frac{\mu_1 \cdot \mu_3}{(4 \cdot \pi \cdot \varepsilon_0) \cdot r^3} \right)$; $D = \frac{3 \cdot \pi \cdot n \cdot \alpha_1 \cdot \alpha_3 \cdot (I_1 \cdot I_3)}{2 \cdot 6 \cdot (4 \cdot \pi \cdot \varepsilon_0)^2 \cdot (I_1 + I_3) \cdot r^3}$;

$$C = \frac{2 \cdot \mu_1^2 \cdot \mu_2^2}{3 \cdot (4 \cdot \pi \cdot \varepsilon_0)^2 \cdot k \cdot T \cdot r^6} + \frac{3 \cdot \alpha_1 \cdot \alpha_2 \cdot (I_1 \cdot I_2)}{2 \cdot (4 \cdot \pi \cdot \varepsilon_0)^2 \cdot (I_1 + I_2) \cdot r^6}$$

Equation (1.13) is rather difficult for mathematical treatment, thus for its simplicity, the authors used an assumption that *r* (distance between the molecules), ratios $\varepsilon^2/\varepsilon_y^2$ and $\varepsilon/\varepsilon_y$ are equal to the constant. In this case, the dependency will be defined mainly by the balance of coefficients (A, B, C, D), and it can be analyzed even using MS Excel, as due to the introduction of a new variable $x=1/\varepsilon$, equation (1.13) transforms into a quadric polynomial equation (1.14):

$$b = C - \frac{\varepsilon^2}{\varepsilon_y{}^2} \cdot D \to const \quad if \quad \frac{\varepsilon^2}{\varepsilon_y{}^2} = const; \quad and \quad T = const.$$

$$d = A - \frac{\varepsilon}{\varepsilon_y} \cdot B \to const \quad if \quad \frac{\varepsilon}{\varepsilon_y} = const; \quad and \quad T = const.$$

$$f = dG_{unpred} = const; \quad and \quad T = const.$$

$$\Delta\mu = \frac{1}{\varepsilon^2} \cdot \left(C - \frac{\varepsilon^2}{\varepsilon_y{}^2} \cdot D \right) + \frac{1}{\varepsilon} \left(A - \frac{\varepsilon}{\varepsilon_y} \cdot B \right) \pm dG_{unpred} = \frac{b}{\varepsilon^2} + \frac{d}{\varepsilon} + f = b \cdot x^2 + d \cdot x \pm f \qquad (1.14)$$

In addition, as a conclusion, we have the final and relatively simple form of the mathematical model (if *T=const*), as equation (2):

$$\ln\left(\frac{m_0}{C \cdot V} - 1 \right) = \frac{\Delta\mu}{R \cdot T} = \frac{b}{\varepsilon^2} + \frac{d}{\varepsilon} \pm f \qquad (2)$$

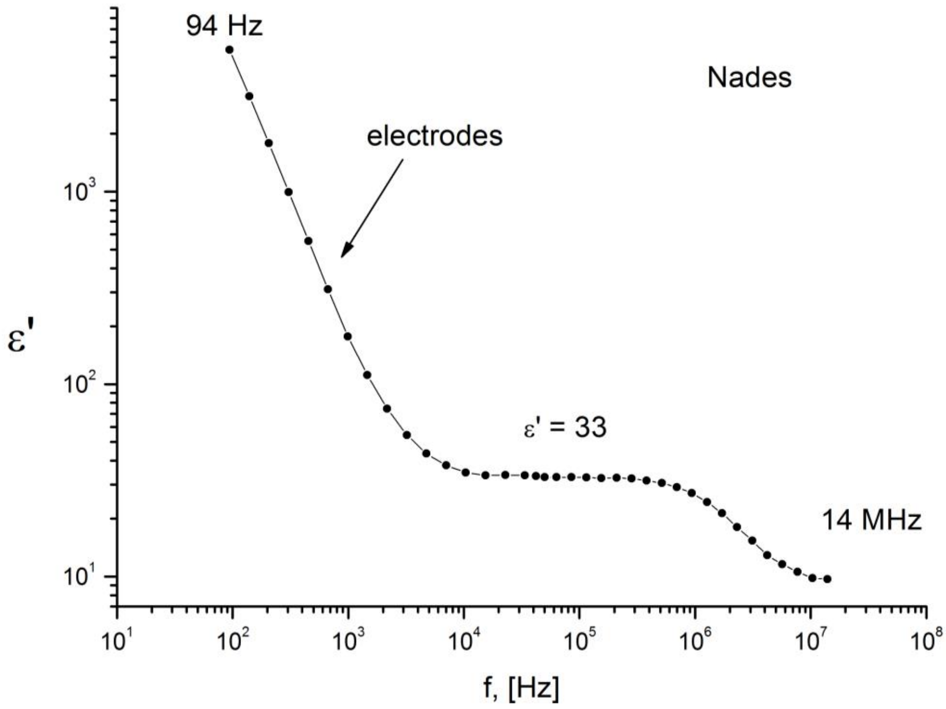

Figure S1: Experimental dependence of a real part of the dielectric constant for sorbitol-based NADES (sorbitol : malic acid : water with molar ratio 1:1:3) on the frequency

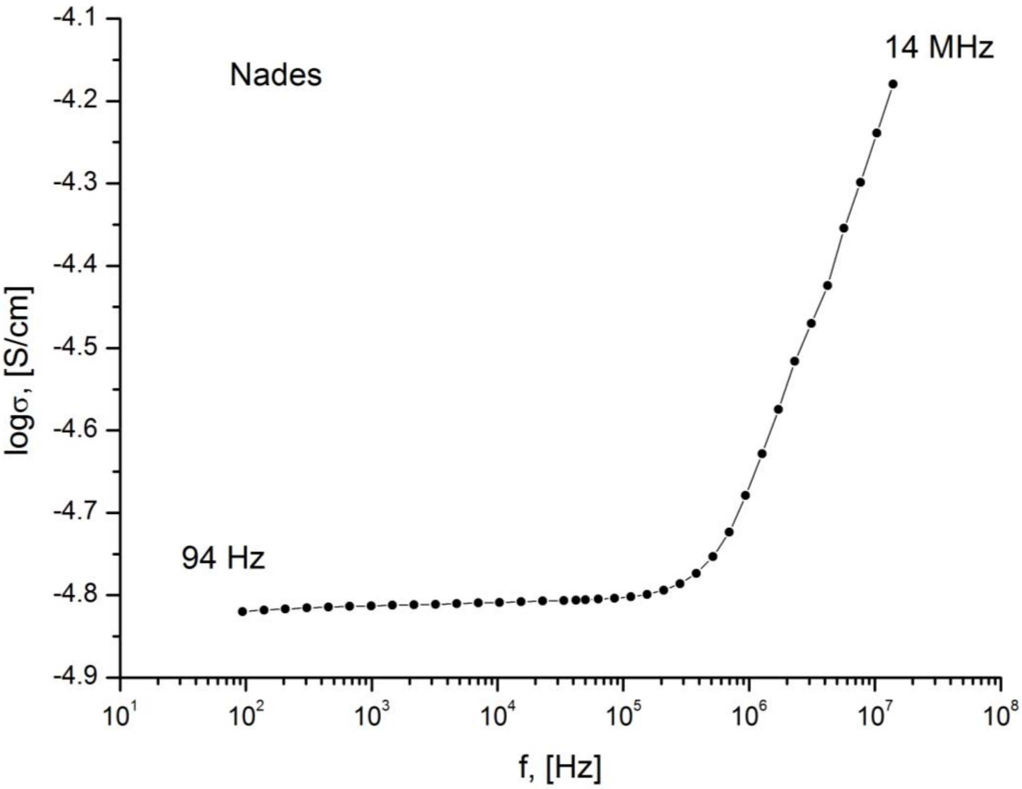

Figure S2: Experimental dependence of the conductivity logarithm for sorbitol-based NADES (sorbitol : malic acid : water with molar ratio 1:1:3) on the frequency

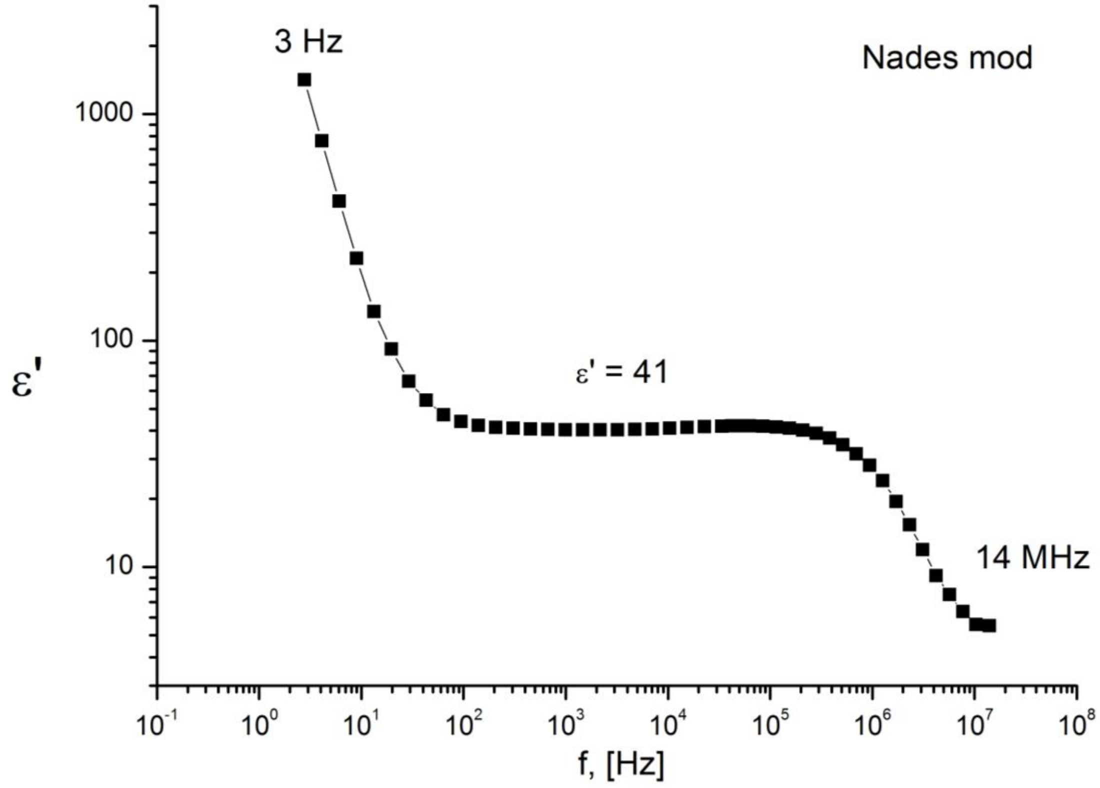

Figure S3: Experimental dependence of a real part of the dielectric constant for modified solvent based on NADES (sorbitol : malic acid : water : glycerin with molar ratio 1:1:1:1) on the frequency

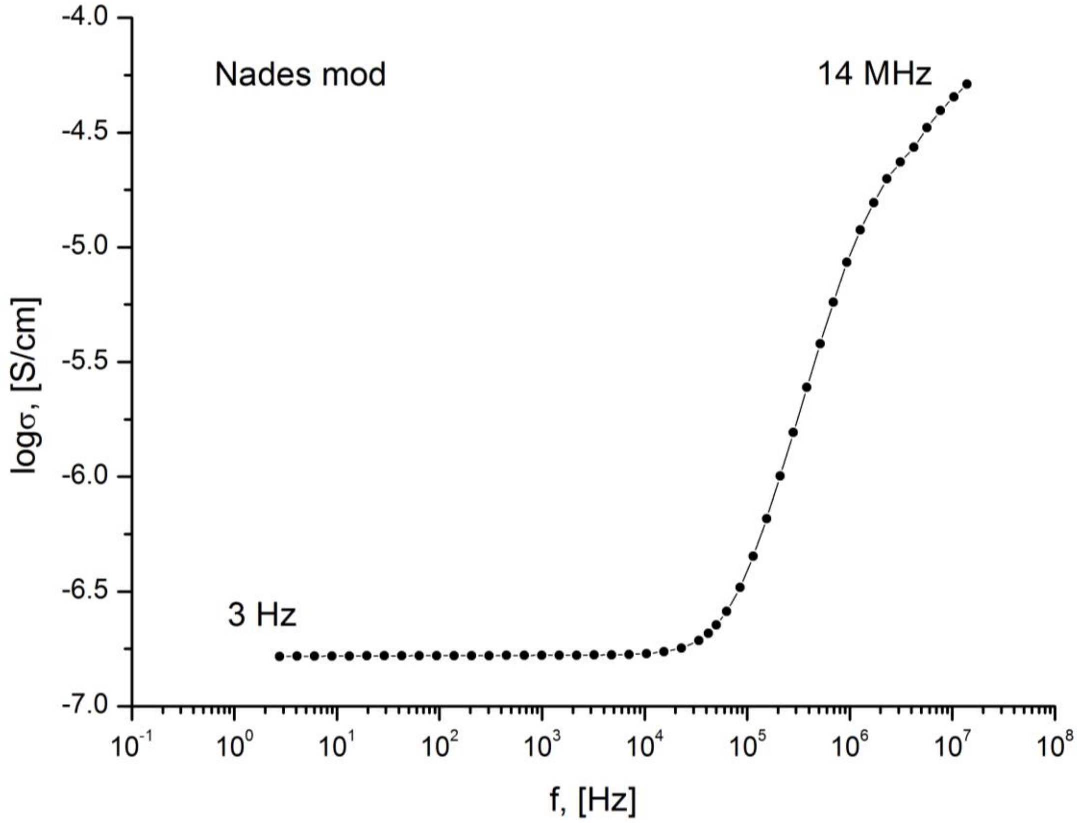

Figure S4: Experimental dependence of the conductivity logarithm for modified solvent based on NADES (sorbitol : malic acid : water : glycerin with molar ratio 1:1:1:1) on the frequency

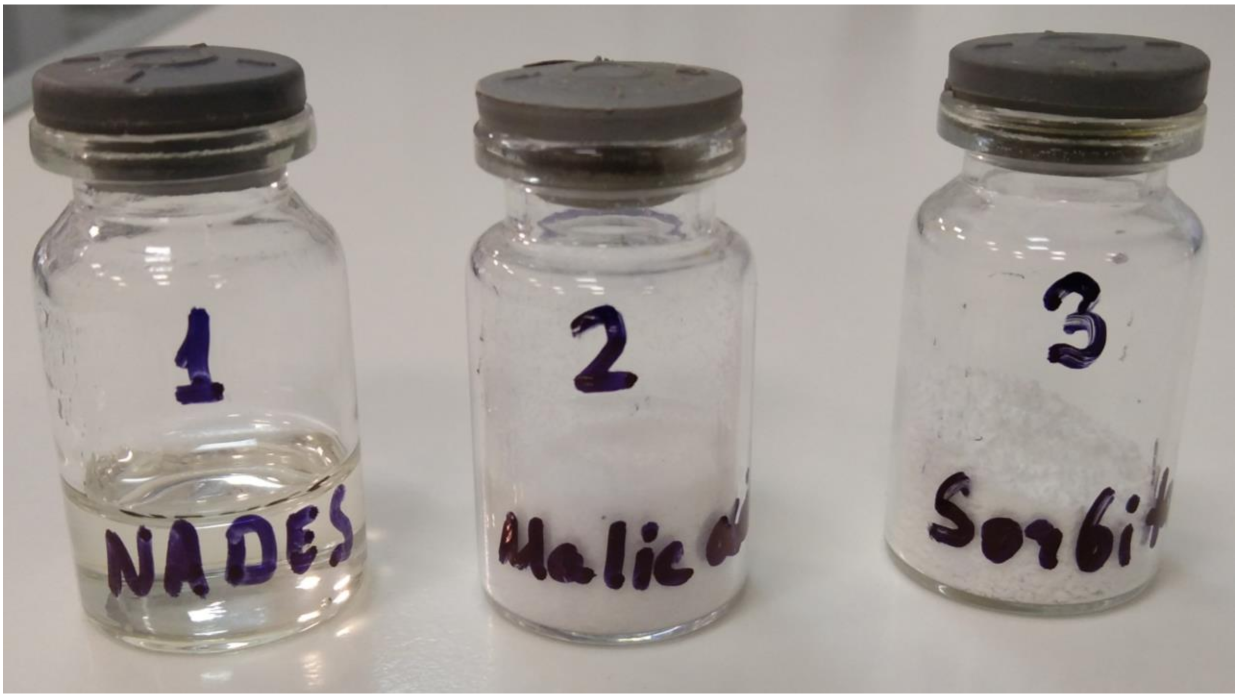

Figure S5: A photo of the physical state of malic acid, sorbitol, and a sorbitol-based NADES (sorbitol : malic acid : water with molar ratio 1:1:3)

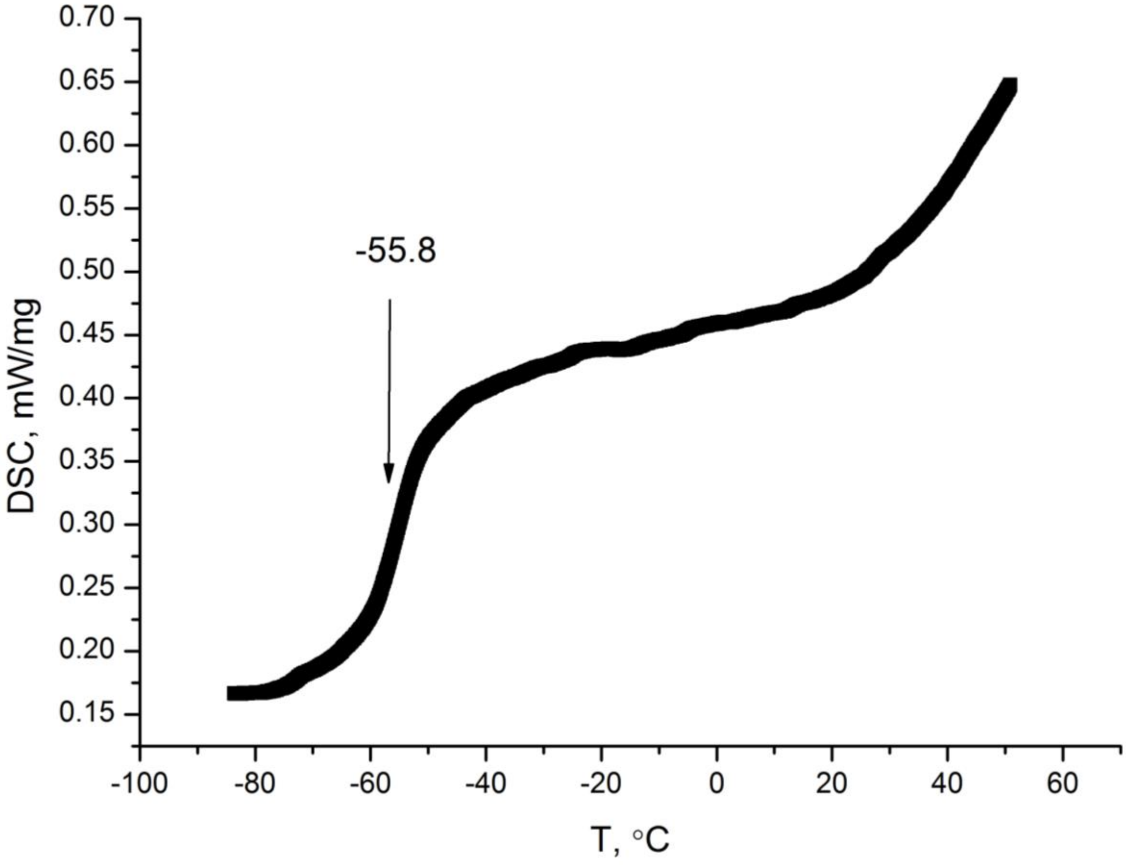

Figure S6: Thermogram for a sorbitol-based NADES (sorbitol : malic acid : water with molar ratio 1:1:3)

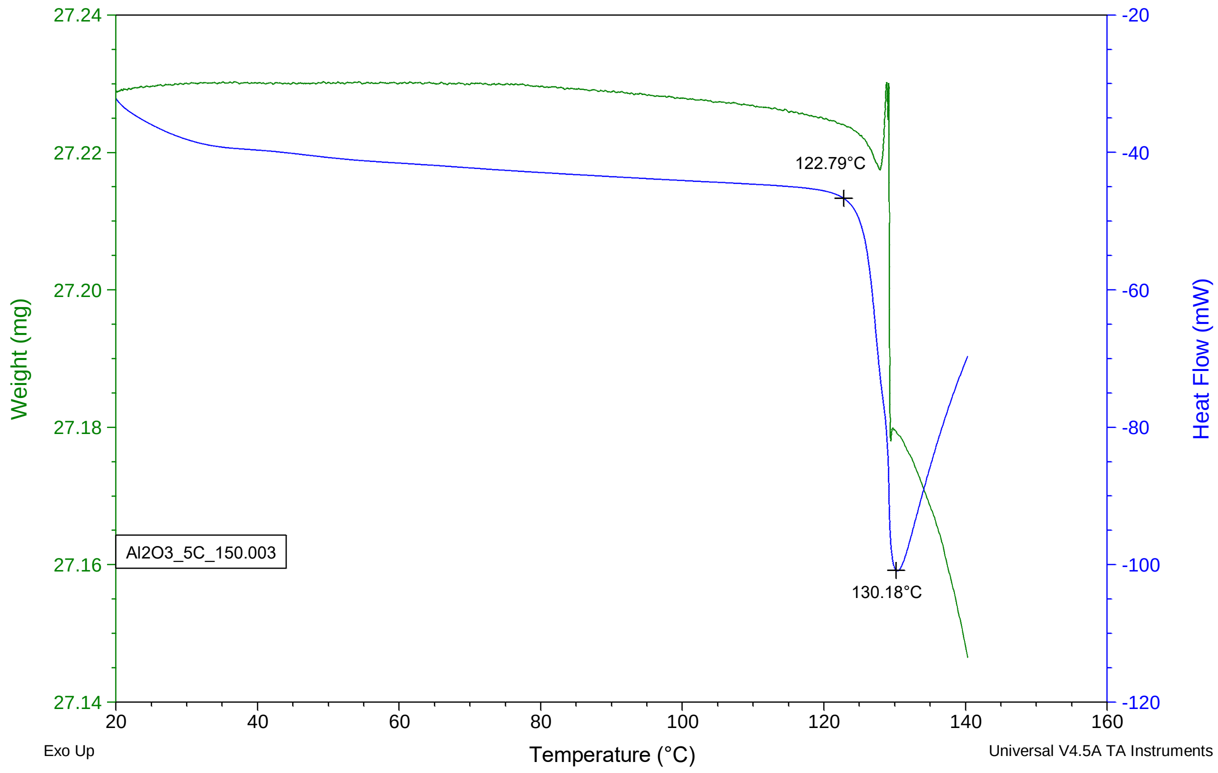

Figure S7: Experimental curves of weight and heat flow dependencies on the temperature for malic acid

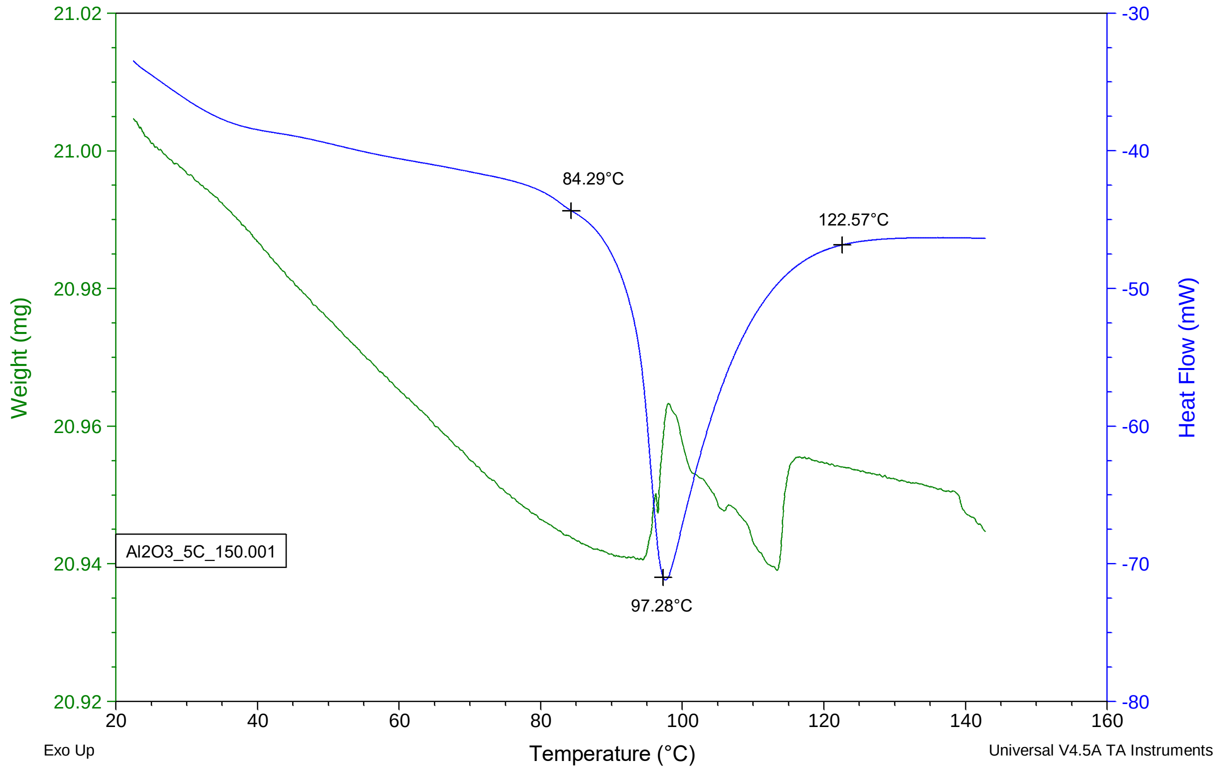

Figure S8: Experimental curves of weight and heat flow dependencies on the temperature for sorbitol

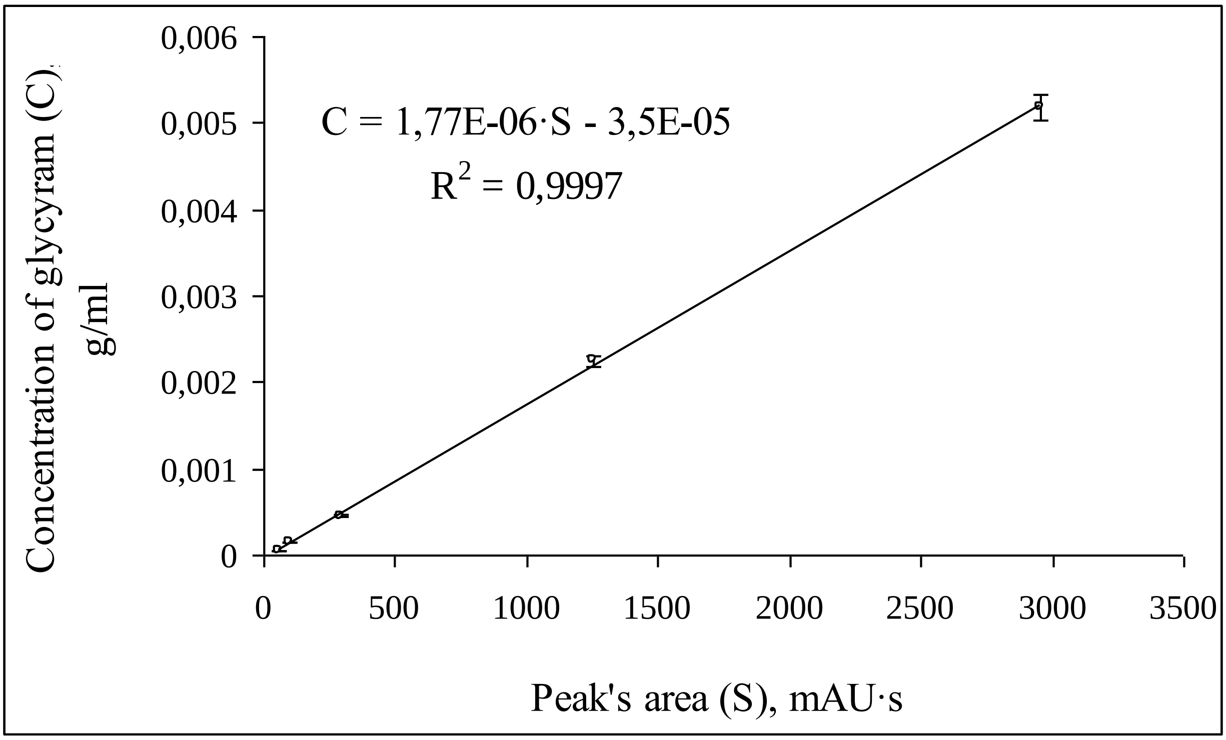

Figure S9: Regression equation of glycyram concentration dependency on the peak's area

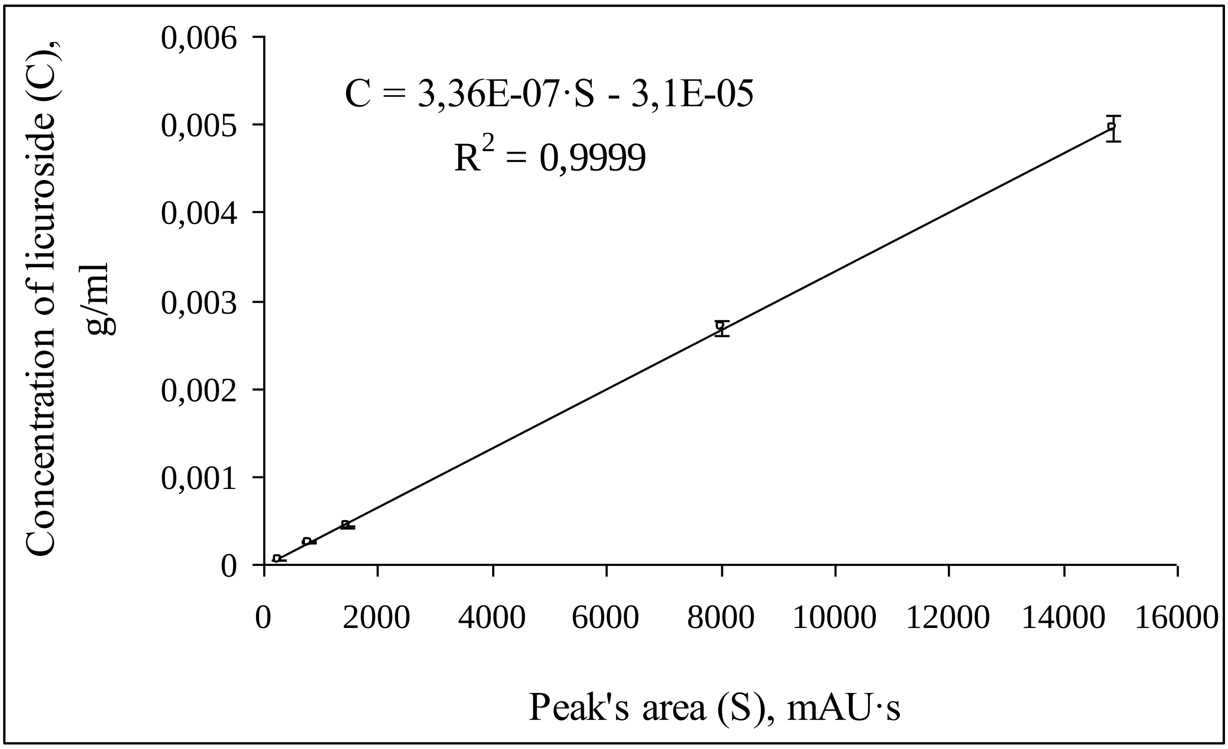

Figure S10: Regression equation of licuroside concentration dependency on the peak's area

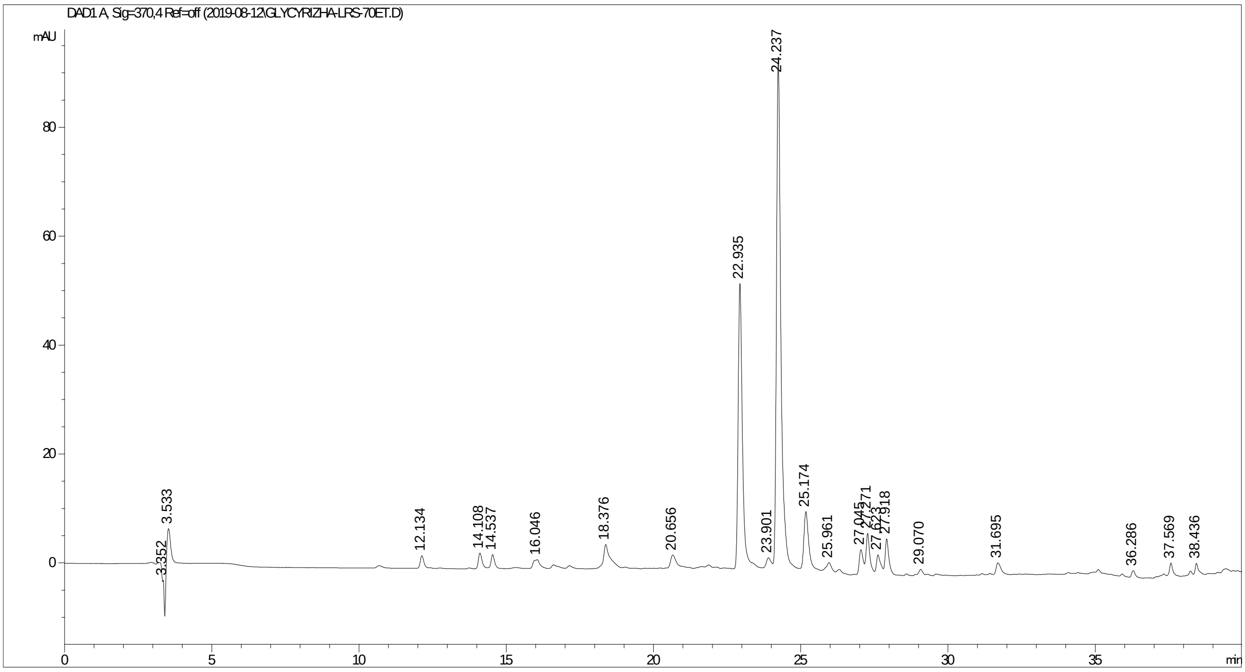

Figure S11: A typical RP HPLC chromatogram of the extract (solvent: ethanol 70% vol., analytical wavelength 370 nm)

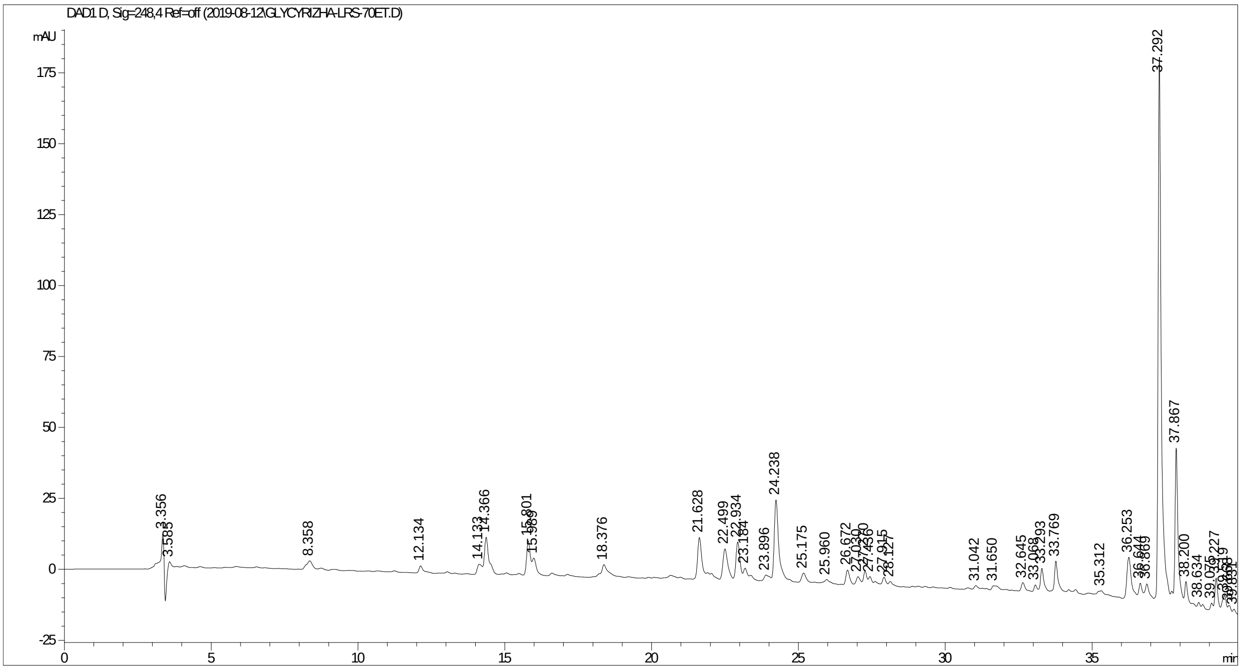

Figure S12: A typical RP HPLC chromatogram of the extract (solvent: ethanol 70% vol., analytical wavelength 248 nm)

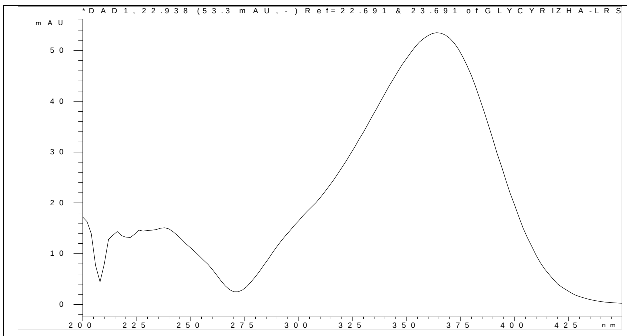

Chalcone 1 (22.9 min)

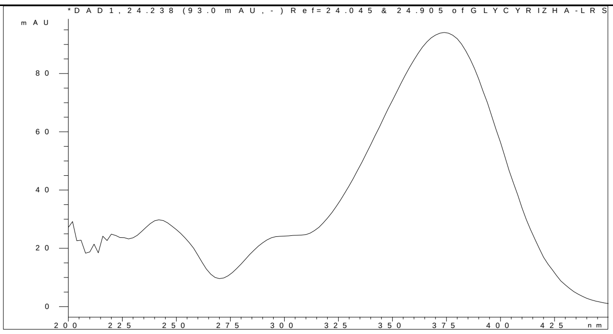

Chalcone 2 (licuroside) (24.2 min)

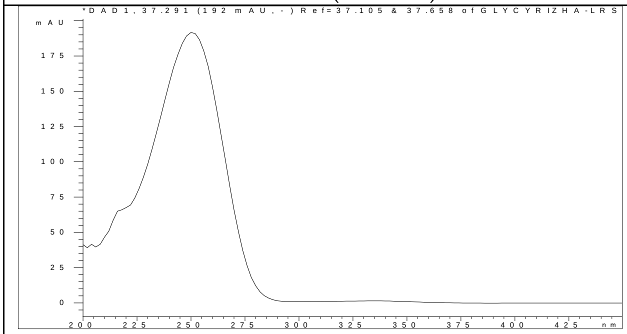

Dominant component of glycyram (37.3 min)

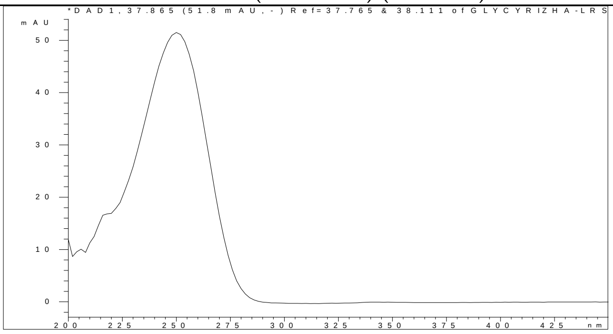

Non-dominant component of glycyram (37.9 min)

Figure S13: Typical UV-spectra of dominant BAS in *Glycyrrhizae* roots (solvent: ethanol 70% vol.)

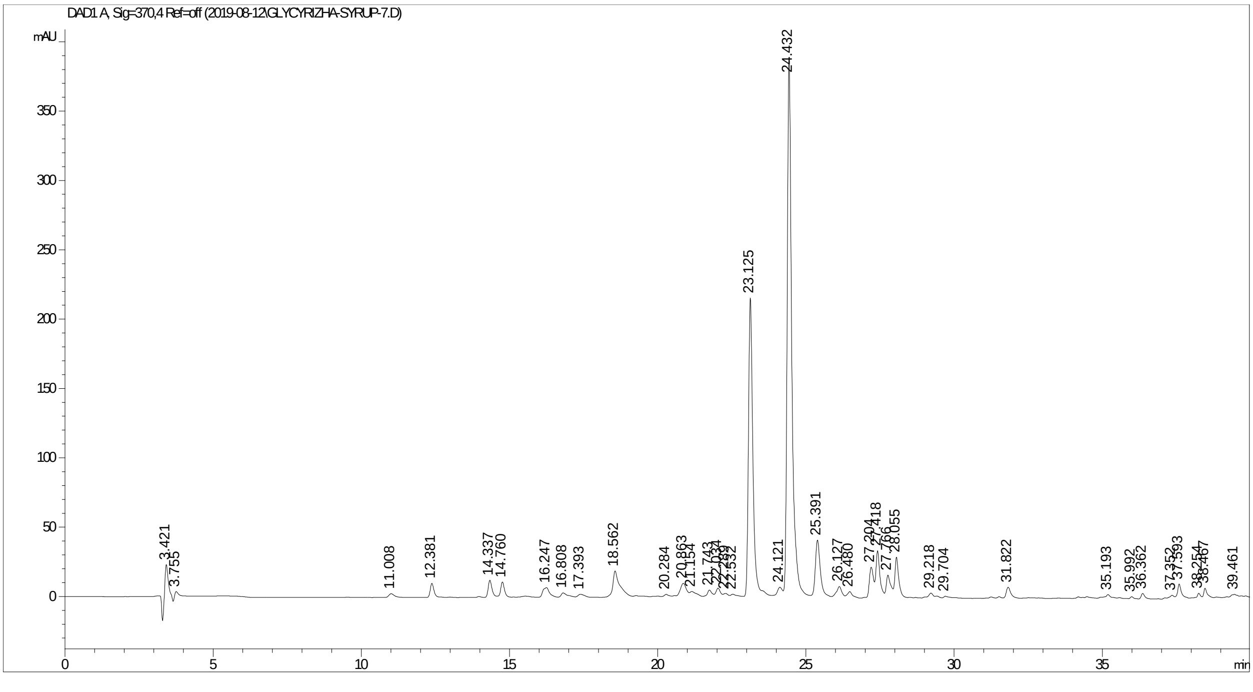

Figure S14: A typical RP HPLC chromatogram of the extract (solvent: sorbitol-syrup, see row 5, table 2, analytical wavelength 370 nm)

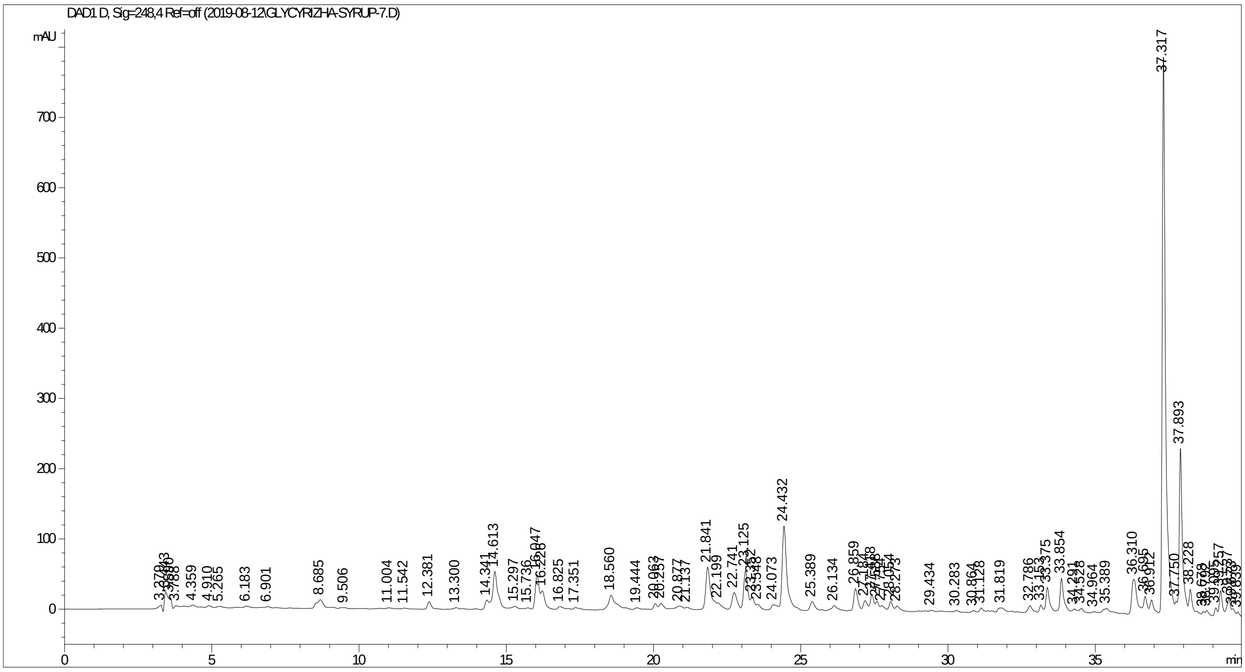

Figure S15: A typical RP HPLC chromatogram of the extract (solvent: sorbitol-syrup, see row 5, table 2, analytical wavelength 248 nm)

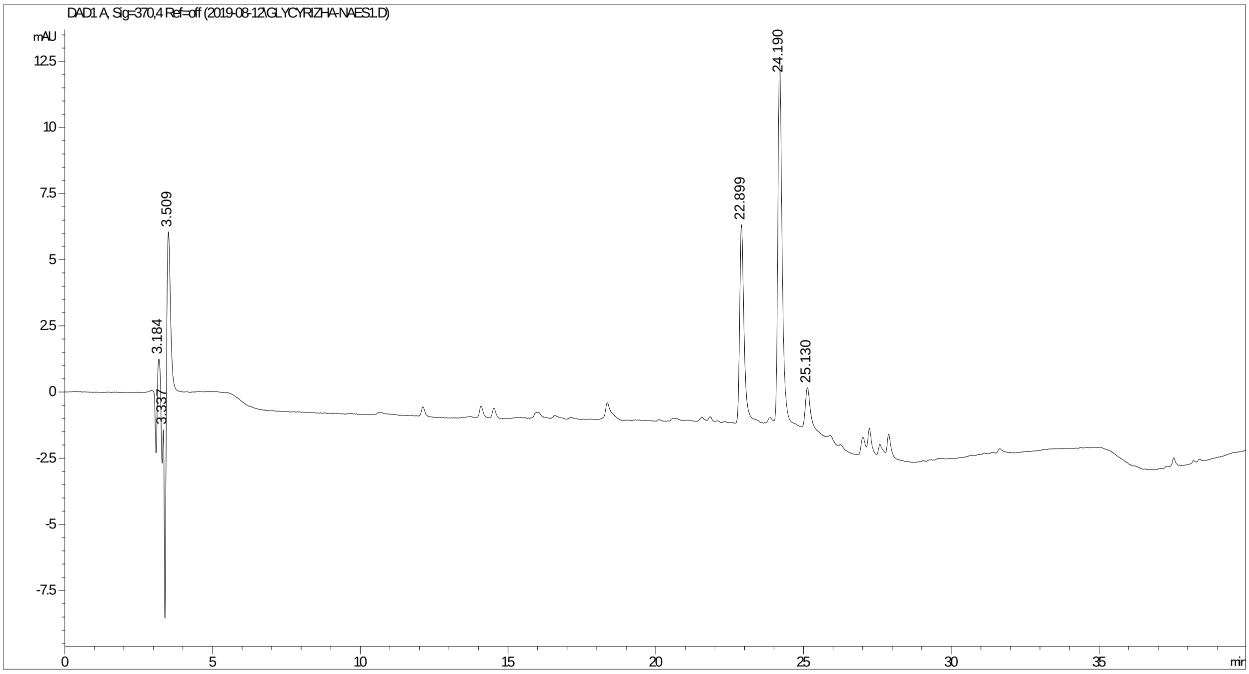

Figure S16: A typical RP HPLC chromatogram of the extract (solvent: NADES, see row 6, table 2, analytical wavelength 370 nm)

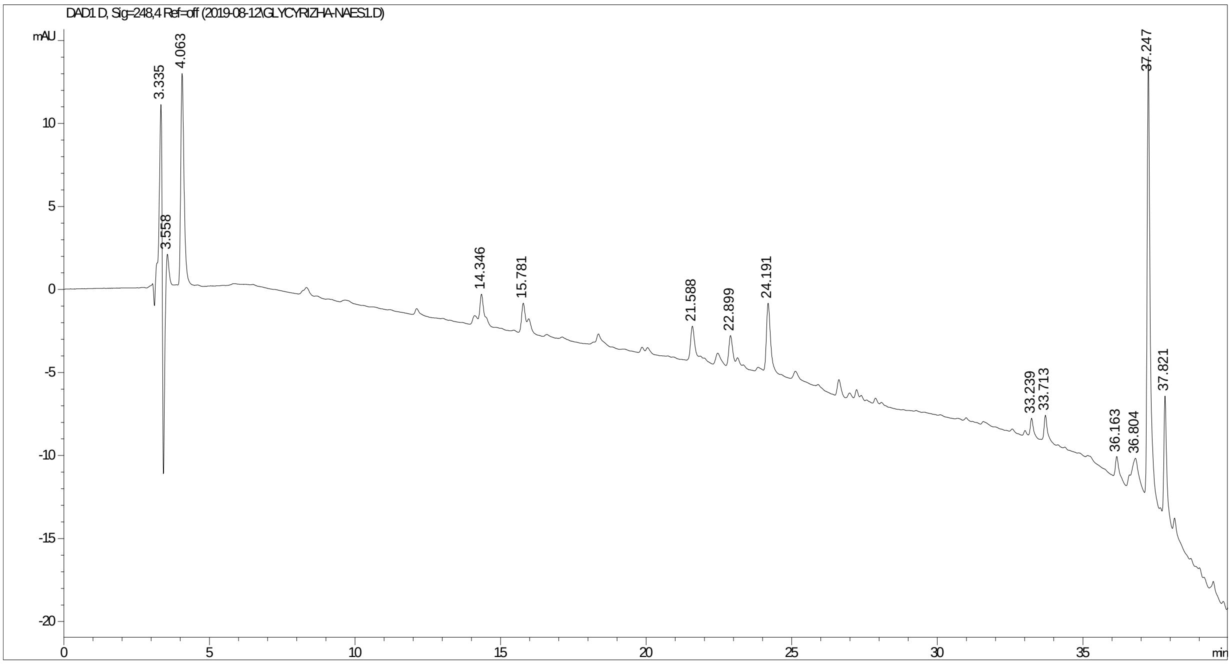

Figure S17: A typical RP HPLC chromatogram of the extract (solvent: NADES, see row 6, table 2, analytical wavelength 248 nm)

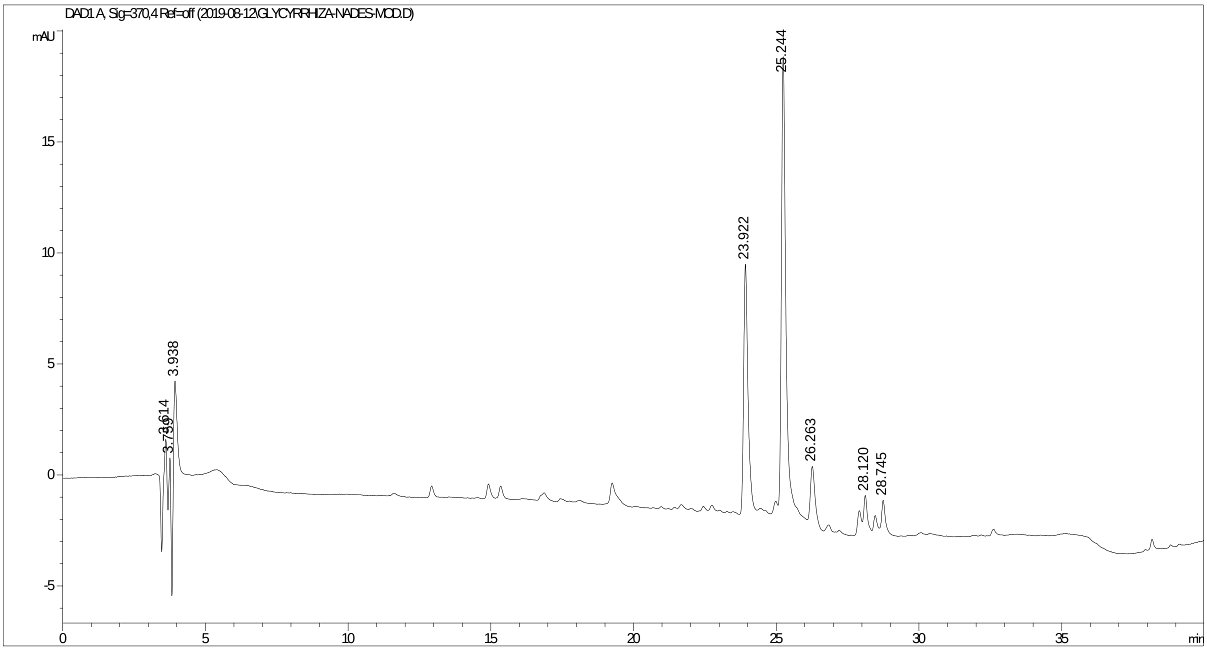

Figure S18: A typical RP HPLC chromatogram of the extract (solvent: NADES-mod, see row 7, table 2, analytical wavelength 370 nm)

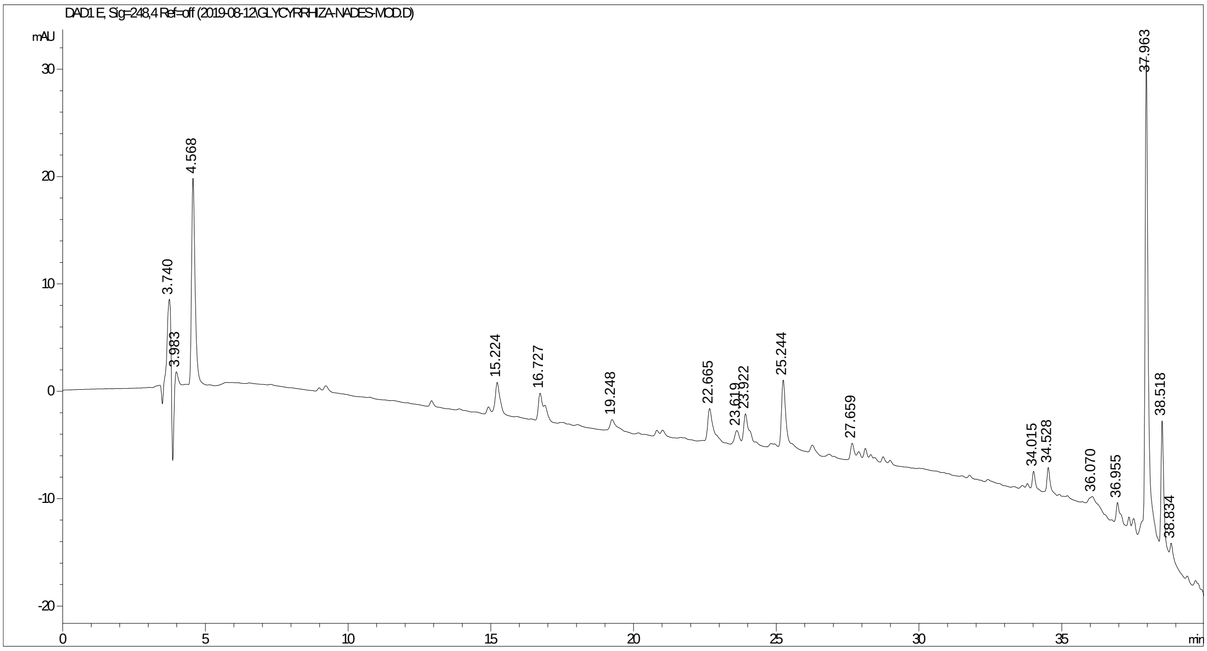

Figure S19: A typical RP HPLC chromatogram of the extract (solvent: NADES-mod, see row 7, table 2, analytical wavelength 248 nm)

Table S1: Data of the extracts' analysis on licuroside and glycyram concentration in them*

No	Weight of plant raw material, g**	Weight of solvent, g	Concentration of licuroside, g/ml	Concentration of glycyram, g/ml	Density of solvent, g/ml
1	1.003±0.005	8.12±0.05	0.00053±0.00003	0.00047±0.00002	0.815±0.006
2	1.007±0.005	8.95±0.06	0.00125±0.00006	0.0102±0.0004	0.900±0.006
3	1.005±0.005	10.42±0.07	0.00126±0.00006	0.0118±0.0005	1.018±0.006
4	1.002±0.005	10.46±0.07	0.00137±0.00007	0.0133±0.0006	1.130±0.006
5	1.004±0.005	12.96±0.08	0.00098±0.00005	0.0102±0.0004	1.298±0.006
6	1.004±0.005	13.80±0.09	0.00113±0.00006	0.0101±0.0004	1.404±0.006
7	1.009±0.005	13.75±0.09	0.00121±0.00006	0.0115±0.0005	1.381±0.006

Note. * Licuroside content in Glycyrrhizae roots was 0.0142±0.0007 g/g, and glycyram content was 0.135±0.007 g/g. ** The mean value and standard error of mean (X±ΔX) were calculated at repeat counts n=3 and significance level P=0.95.

Table S2: Theoretical values of the determination coefficient (R^2)*

Number of experimental points (n)	Level of acceptance probability (P=90)			Level of acceptance probability (P=95)			Level of acceptance probability (P=99)		
	Number of variables in equation (xi)								
	1	2	3	1	2	3	1	2	3
3	0.976	-	-	0.994	-	-	1.000	-	-
4	0.810	0.990	-	0.902	0.997	-	0.980	1.000	-
5	0.649	0.900	0.994	0.771	0.950	0.998	0.919	0.990	1.000
6	0.532	0.785	0.932	0.658	0.864	0.966	0.841	0.954	0.993

Note. * The source: Baraz, V.R., Pegashkin, V.F. Using the MS Excel for the statistical analysis of data. Nizhniy Tahil: NTI (filial) UrFU, 2014. (In Russ.)
